# Acetaminophen poisonings in Chilean healthcare settings: a 20-year story that does not end

**DOI:** 10.3126/nje.v11i4.38919

**Published:** 2021-12-31

**Authors:** Tamara Sanhueza-Aroca, Samuel Verdugo-Silva, Erwin Olate-Fica, Luisa Rivas, Claudio Müller-Ramírez

**Affiliations:** 1-3 Independent consultant. Concepcion, Chile; 4 School of Physical and Mathematical Sciences, University of Concepcion, Chile; 5 School of Pharmacy, University of Concepcion, Chile

**Keywords:** Epidemiology, Over-the-counter drugs, Suicide, Toxicology

## Abstract

**Background:**

Acetaminophen (Paracetamol) is one of the most used and prescribed anti-inflammatory and analgesic drugs worldwide. It has become one of the main drugs related to accidental and intentional overdoses in many countries, including Chile. The objective of this work was to characterize acetaminophen poisonings occurred in Chile between the years 2001 and 2020.

**Methods:**

A retrospective study of acetaminophen poisonings among patients who were hospitalized in Chilean public and private hospitals was carried out between the years 2001 and 2020. Data was obtained from the Medical Outcome Statistical Report database. Inclusion criteria were cases of patients who were admitted into either public or private healthcare settings with diagnosis of acetaminophen poisoning according to the WHO ICD-10. Statistical analyses were run to establish associations between variables selected in the study.

**Results:**

A total of 2,929 cases were included in the study. 77 % of the cases corresponded to female patients (p<0.05). Patients’ age range went from 0 to 81 years old. Adolescents and young adults resulted more involved in reported cases during the 2001-2020 period (p<0.001). During the first period of the study, accidental poisonings were more commonly reported, however in the recent years intentional cases increased their occurrence, especially among female patients (p<0.05). A multivariate logistic regression model considered as statistically significant (p<0.05) the interaction between the variables age, gender and year of the event.

**Conclusion:**

The present study identified a large number of acetaminophen poisonings reported in Chile during the 2001-2020 period. Cases were characterized including patients’ gender, age, and poisoning intent. Health authorities should consider these findings as an opportunity to improve public health associated with the use and misuse of over-the-counter drugs, including acetaminophen.

## Introduction

Self-poisonings may result from exposures to almost any chemical (e.g. drugs, cosmetics, gases) and they can be classified into accidental and intentional episodes [1]. Drug overdose is a common cause of self-poisonings in several regions around the globe [2].

Acetaminophen (Paracetamol) is one of the most prescribed anti-inflammatory and analgesic drugs worldwide. It is also considered as a safe medication when is used within the therapeutic range (i.e. 1-4 g/day) [3]. However, acetaminophen has the potential of causing intrinsic and dose-dependent hepatotoxicity [4]. Clinical complications for patients go from reversible liver injury through liver transplant [5]. As a matter of fact, acetaminophen overdoses are associated with the highest rates of liver failure in the United States of America (USA) [6].

Particularly, acetaminophen is an inexpensive over-the-counter drug, which poses high pharmacology efficacy. These characteristics have made it widely available on the market and it has been related to accidental and intentional overdoses in many countries. Specifically, acetaminophen overdoses represent 5 % of ED visits in the USA, and an average of 60,000 patients are admitted into hospitals each year [7].

In Chile, acetaminophen is the second most consumed drug as to its analgesic properties in acute and chronic medical treatments [8]. National epidemiological information about poisonings related to acetaminophen is scarce and limited. To our knowledge, there are two publications associated with acetaminophen poisoning trends, the first is based on information from a one-year report of phone calls received at one of the local Poison Control Centers in the year 2009 [9], and the second publication corresponds to a retrospective study conducted in a large Chilean public hospital between the years 2008 and 2010 [10]. These works provide a time-limited overview of national trends of acetaminophen accidental and intentional poisonings, which not necessarily may represent the current scenario. More recent data is needed to better understand national patterns of acetaminophen overdose episodes.

The objective of this work was to characterize acetaminophen poisonings occurred nationwide between the years 2001 and 2020.

## Methodology

### Study design and participants

A retrospective study of acetaminophen poisonings related to patients who were admitted into Chilean public or private hospitals was carried out between the years 2001 and 2020.

### Data collection

Data was obtained from the Medical Outcome Statistical Report (MOSR) open access database, which is released yearly by the Department of Statistics and Health Information of the local Ministry of Health. It basically compiles all medial discharge reports associated with patients admitted into either public or private healthcare settings in Chile. Information contained in the MOSR database between the years 2001 and 2020 was filtered by considering the inclusion and exclusion criteria. Consequently, all cases that fell into the inclusion criteria were tabulated on a Microsoft Excel® spreadsheet to facilitate data processing.

### Inclusion criteria

Inclusion criteria were cases of patients who were admitted into either public or private healthcare settings with clinical diagnosis of acetaminophen poisoning according to the World Health Organization International Classification of Diseases ICD-10. Thus, a series of 3 codes associated with acetaminophen poisonings were selected: T39.1 (4-aminophenol derivatives poisoning), X40 (accidental 4-aminophenol derivatives poisoning), and X60 (intentional 4-aminophenol derivatives self-poisoning) [11].

### Exclusion criteria

Neither code Y10 (poisoning by and exposure to nonopioid analgesics, antipyretics and antirheumatics, undetermined intent, including 4-aminophenol derivatives), nor code Y45.5 (4-aminophenol derivatives causing adverse effects in therapeutic use) were considered [11].

### Sample size calculation

This work is based on a population study related to patients diagnosed with acetaminophen poisoning along the country, therefore there was no sample size calculation.

### Outcome variable

The main outcome variable was the characterization of acetaminophen poisoning trends among Chilean population.

### Explanatory variable

Selected data was sorted by taking into account the following variables: patient’s gender, age, and poisoning intent (accidental and intentional exposure to acetaminophen, X40 and X60 ICD-10 respectively).

Patients’ age classification was considered as follows: newborn/toddler >0 < 2, preschool ≥2 ≤ 5, school ≥ 6 ≤ 10, adolescents ≥11 ≤ 17, young adults ≥18 ≤ 35, adults ≥ 36 ≤ 64, and elder ≥ 65 years old.

As for poisoning intent, accidental and intentional events were associated with exposure to/ingestion of supratherapeutic doses of acetaminophen without the intention of self-harm, and with the intention of self-harm respectively [12].

### Ethical committee approval

Information collected for this study was obtained from an open data base, which keeps patients’ sensitive information (e.g. name, social security number, address) confidential at all times. Also, declaration of Helsinki was adhered to. Consequently, no ethical committee approval was required to conduct this work.

### Data management and statistical analysis

Statistical analysis was carried out by using the software R-project version 3.6.1 (R Core Team 2013).

Categorical data is presented as absolute frequencies and percentages. Comparison among categorical variables was made by employing the Pearson chi-square and exact Fischer’s tests; this was considering the usual condition that 80% of expected values (Ei) ≥ 5. Also, comparison of proportions was carried out by using the one-sided proportion test.

As for continuous data, descriptive statistics was utilized (e.g. median, range). The medians were compared by using either the non-parametric Mann-Whitney U-test or the Wald Wolfowitz test according to previous verification of homoscedasticity, which was determined by the Flinger test. Also, Odd Ratios were calculated.

Additionally, multivariate logistic regression was used to analyze predictors of acetaminophen poisonings where event intent (intentional/accidental) was the dependent variable. A p-value < 0.05 was considered statistically significant.

## Results

### Demographic characteristics of patients:

The study identified 2,929 cases associated with hospital inpatient discharges with acetaminophen poisoning (T39.1 ICD-10) during the 2001-2020 period. Table 1 shows nationwide gender distribution among patients admitted into either public or private healthcare settings with clinical diagnosis of acetaminophen poisoning.

Female patients were related to 77% of cases while male patients represented 23% of the total registered events along the period. The proportion of female patients with clinical diagnosis of acetaminophen poisoning is statistically larger than that of males patients (p<0.05), except for year 2004 (p=0.0888). Additionally, there was no statistically significant difference among all cases reported during the period when comparing each year to its predecessor. This confirms that female patients were more frequently involved in acetaminophen poisonings than male patients.

According to the results obtained from the one-sided proportion test for the year 2004 (Table 1), a Pearson`s proportion homogeneity test was run. A p-value < 0.05 was obtained. Thus, the study period was grouped by years (i.e. Group 1 2001-2007, Group 2 2008-2013, and Group 3 2014-2020).

According to Table 2, each group presents a larger proportion of female patients when compared to that of male patients (p < 0.001). In addition, there are statistically significant differences among groups when considering patients’ gender. This is when contrasting groups 1 to 2 (p < 0.020), and 1 to 3 (p < 0.003).

Also, age ranges of poisoned patients were between 0 and 81 years old considering the entire period of study. As for female patients, during the 2008-2013 period (group 2), the age range decreased when compared to groups 1 and 3. In contrast, the age range of male patients suffered small variations when comparing the three groups. Also, there were statistically significant differences for the variable age (expressed as median) when comparing patients’ gender. These results are applicable within and between the groups (p-value < 0.05).

From Table 3, it can be established that there are statistically significant differences among the 3 groups (p < 0.001). Particularly, adolescents in groups 2 and 3 relate to the highest percentage of acetaminophen poisonings (i.e. 37.4% and 48.3% respectively), while young adults are associated with this phenomenon in group 2 (i.e. 40.2%). Also, frequencies of poisoned preschool patients decreased over the years.

In group 1, overall accidental acetaminophen poisonings were more frequently encountered (i.e. 220) when contrasted to intentional events (i.e. 185). Female patients were more involved in both, accidental and intentional poisonings than male patients (i.e. 290 vs 115). In groups 2 and 3, there were more intentional acetaminophen poisonings than accidental events (i.e. 2,167 vs 357), this is regardless the gender (p-value < 0.001). Also, female patients, in the three groups, were more associated with intentional poisonings than male patients (Gender vs Intent p-value < 0.05).

Furthermore, when comparing proportions of accidental and intentional acetaminophen poisonings between groups, there are statistically significant differences between groups 1 and 2, groups 1 and 3; and groups 2 and 3 (p-value < 0.05).

Additionally, odd ratios (OR) of each group were calculated to estimate probabilities of acetaminophen poisonings occurrence. Thus, OR for group 1 was 2.083, this means that the ratio among intentional vs accidental acetaminophen poisonings is 2.083-fold higher among female than that in male patients. ORs for groups 2 and 3 were 2.638 and 2.465 respectively.

The multivariate logistic regression model considered the dichotomous variable event intent as the dependent variable (i.e. intentional event = 1 or accidental event = 0), which was denoted as Y. Also, this model included the following independent patient variables denoted as X: age, gender, and group.

The independent variable group is not statistically significant when considered alone. However, when this variable is considered along with the age, there is statistically significant interaction in terms of acetaminophen poisoning occurrence. Similarly, the variable gender (i.e. female patient), this condition is significant to the regression model.

## Discussion

Acetaminophen is commonly used in most of the countries around the world where it is available as both an over-the-counter and a prescribed medicine. Acetaminophen overdoses can cause not only liver, but also kidney toxicity among poisoned patients.

Most of the acetaminophen overdose episodes have been related to adolescents and young adults. In both cases, mental health problems among patients are a common clinical finding (e.g. depression, Attention Deficit Hyperactivity Disorder) [13].

### Acetaminophen poisoning rates

According to this study, acetaminophen poisoning rates increased along the 2001-2020 period. Particularly, the highest value was achieved in 2020 (i.e. 22.5 per 100,000 hospital admittances).This phenomenon has also happened in other countries such as Spain (2005 to 2010) [14], Australia (2004-2017) [15], and the USA (1998-2008) [16]. In contrast, Canada has shown a permanent decrease of these rates during the 1998-2004 [17] and 2011-2019 periods [18]. Likewise, the USA has achieved lower rates between the years 2008 to 2011 [16]. This phenomenon is not completely clear, but it can be explained by the introduction of warning labels in pharmaceutical products containing acetaminophen.

Considering the above information, an average of 145 ± 85 patients, including both genders, were nationwide admitted into either public or private healthcare settings with clinical diagnosis of acetaminophen poisoning. Of these, female patients were more associated with acetaminophen poisonings along the entire period of study. This scenario has also been reported in the work of Kaur et al. [18].

### Age and gender of poisoned patients

Adolescent patients were involved in at least 73% of intentional acetaminophen poisonings. Of these cases, most events were associated with adolescent female patients who ingested this drug with self-harm purposes. Young adults were involved in at least 48% of all cases. Similarly to what is described above, at least 60% of these cases corresponded to female patients. These findings are similar to those reported in the other works [7,12,15,19,20].

Considering all acetaminophen poisonings (2001-2020 period) grouped into the three groups, a clear trend in terms of increases in the number of events over the years was noticed. Group 1 corresponded to the events occurred between the years 2001 and 2007 where 405 events took place. Groups 2 and 3 showed more notorious increases with 882 and 1,642 cases respectively over the 2008-2020 period. These findings may be explained by the wide availability of acetaminophen contained, as a single drug and in combination with other drugs, in marketed drug products, especially in the most recent years [21-24].

When including patients’ gender, there were statistically significant differences when comparing Group 1 to Group 2 and Group 1 to Group 3. However, there was no statistically significant difference when comparing the last two groups pointing out that the proportion of poisoned patients of both genders remained similar.

Complimentary, these kinds of poisonings affected patients of all ages. In the present work, we found an overall range of patients’ ages similar to the other works [25,26]. On one hand, male patients showed a notorious increase in terms of age median from group 1 to group 2. On the other hand, female patients showed a small variation from group 1 to group 2. For both genders, there was no variation during the last period of the study (group 3). This confirms that Chilean female patients have shown a common pattern of exposure to acetaminophen for two decades.

As for age category of poisoned patients, adolescents and young adults resulted more frequently involved in these events. This pattern has also been reported in the work of Tan et al. [27]. Also, we observed a decrease in the number of events where preschool patients were involved. This situation is consistent with the work of Nistor et al., [28] and Parry et al. [29].

### Poisoning intent

In regards with poisoning intent, during the first 7 years of the study (i.e. Group 1), overall accidental acetaminophen poisonings were more frequently reported. This is also shown in a study conducted in the Italian North-West area [30]. However, in Groups 2 and 3, the last 13 years of the study, overall intentional acetaminophen poisonings increased. This trend has also been reported by Piotrowska et al. [31].

When considering patients’ gender and poisoning intent, female patients presented the largest number of both intentional and accidental acetaminophen poisonings during the 2001-2020 period. These trends have also been described in the works of Casey et al., (i.e. 34.3 % of female and 30.5 % of male patients) and Cairns et al., (i.e. 71.6 % of female and 28.4% of male patients) [15,32]. Particularly for intentional events, female patients presented higher ORs of acetaminophen poisoning occurrence when compared to male patients.

As for the information obtained from the multivariate regression model, it is important to consider that the independent variables age, group and gender, particularly being a female patient, showed significant interaction in terms of predicting the occurrence of acetaminophen poisonings.

Furthermore, patients admitted into healthcare settings because of intentional poisonings should receive immediate psychiatric attention, especially those who repeatedly present with suicide attempts. This way is easier to predict common patters among these types of individuals who have previously shown self-harm behavior [33]. Also, other intentional poisoning risk factors are associated with alcohol or illicit drug consumption, and psychological stressors (e.g. social isolation, family conflicts) [13].

Finally, considering the variables analyzed and discussed in this work is of high importance at the moment of designing and implementing poisoning prevention strategies [34,35].

## Limitation of the study

As of the year 2007, the local Ministry of Health mandated by means of the Medical Outcome Statistical Report act, that either public or private healthcare institutions must provide information about all patient admittances associated with any diagnosed pathologies, including poisonings and drug adverse effects [36]. This scenario turns into a limitation of the study since acetaminophen poisoning events occurred between the years of 2001 and 2006 were not linked to the Medical Outcome Statistical Report act. Thus, the number of poisonings within this period may have been underestimated. Also, this condition creates database heterogeneity, which may influence data interpretation.

Another limitation of the study is that related to acetaminophen poisonings that were resolved at emergency departments and did not require patient hospitalization. This situation may have also contributed to underestimation of poisonings.

## Conclusion

The present study identified a large number of acetaminophen poisonings reported in Chile during the 2001-2020 period. Poisoning events were initially characterized including patients’ gender, age, and poisoning intent (accidental or intentional), according to the WHO’s ICD-10. Female patients resulted more frequently involved in the events regardless poisoning intent. Also, adolescents and young adults were related to the largest number of poisonings along the period. Furthermore, statistical associations between the studied variables were made. Health authorities should pay special attention to these kinds of poisoning events since there are many aspects involved in these clinical scenarios that may be taken as an opportunity to improve public health associated with the use and misuse of therapeutic drugs, including the incorporation of warning labels in over-the-counter pharmaceutical products such as acetaminophen, educational campaigns focused on target populations (e.g. children, female adolescents, and young adults), and increasing accessibility to mental health programs.

### Future scope of the study

A more comprehensive study, including more recent information of acetaminophen poisonings is planned. Also, this study will consider adding other drugs and non-drug agents, which are commonly found in poisoned patients.

### What is already known on this topic?

In Chile, the available information about acetaminophen poisoning trends among local population is scarce and needs to be updated.

### What this study adds

Information related to epidemiological trends in terms of acetaminophen poisonings in Chilean population. This data can be used to create and implement prevention strategies and also to better train medical personnel to provide patients with appropriate treatments.

## Figures and Tables

**Table 1:** Yearly distribution of acetaminophen poisonings by patients’ gender

Year	Male (%)	Female (%)	Male vs Female p-value*	Year p-value**
2001	6 (27.3)	16 (72.7)	0.0275	
2002	9 (22.5)	31 (77.5)	0.0004	2001 vs. 2002: 0.9124
2003	7 (25.9)	20 (74.1)	0.0105	2002 vs. 2003: 0.9757
2004	22 (40.0)	33 (60.0)	0.0888	2003 vs. 2004: 0.3139
2005	25 (27.8)	65 (72.2)	<0.0001	2004 vs. 2005: 0.1793
2006	17 (23.6)	55 (76.4)	<0.0001	2005 vs. 2006: 0.6738
2007	29 (29.3)	70 (70.7)	<0.0001	2006 vs. 2007: 0.5140
2008	18 (17.3)	86 (82.7)	<0.0001	2007 vs. 2008: 0.0633
2009	19 (21.6)	69 (78.4)	<0.0001	2008 vs. 2009: 0.5713
2010	36 (28.6)	90 (71.4)	<0.0001	2009 vs. 2010: 0.3217
2011	25 (18.5)	110 (81.5)	<0.0001	2010 vs. 2011: 0.0765
2012	39 (20.7)	149 (79.3)	<0.0001	2011 vs. 2012: 0.7237
2013	59 (24.5)	182 (75.5)	<0.0001	2012 vs. 2013: 0.4244
2014	47 (23.7)	151 (76.3)	<0.0001	2013 vs. 2014: 0.9448
2015	50 (24.9)	151(75.1)	<0.0001	2014 vs. 2015: 0.8363
2016	35 (19.2)	147 (80.8)	<0.0001	2015 vs. 2016: 0.2065
2017	35 (16.4)	179 (83.6)	<0.0001	2016 vs. 2017: 0.5383
2018	52 (18.4)	231 (81.6)	<0.0001	2017 vs. 2018: 0.6402
2019	61 (21.3)	225 (78.7)	<0.0001	2018 vs. 2019: 0.4365
2020	71 (25.5)	207 (74.5)	<0.0001	2019 vs. 2020: 0.2795
Total	662 (22.6)	2,267 (77.4)		
N= 2,929				

* One-sided proportion test

** Pearson’s chi-square test (two-sided)

**Table 2:** distribution and statistical significance of acetaminophen poisonings according to patients’ gender and age

	Group 1^a^		Group 2^b^		Group 3^c^		G1 vs G2 p-value	G1 vs G3 p-value	G2 vs G3 p-value
Gender	N (%)	Age median (range)	N (%)	Age median (range)	N (%)	Age median (range)			
**Male**	115 (28.4)	5 (0-76)	196 (22.2)	18 (0-58)	351 (21.4)	18 (0-74)	0.020^e^	0.003^e^	0.659^e^
**Female**	290 (71.6)	16 (0-74)	686 (77.8)	17 (0-81)	1,291 (78.6)	17 (0-79)	<0.001^g^	<0.0001^f^	0.9861^g^
**Total**	405 (100.0)	15 (0-76)	882 (100.0)	17 (0-81)	1,642 (100.0)	17 (0-79)	<0.001^g^	<0.001^f^	<0.001^f^
**M vs F** **p-value**	<0.001^d^	<0.001^f^	<0.001^d^	<0.001 ^f^	<0.001^d^	<0.001^f^			
**N= 2,929**									

a: 2001-2007

b: 2008-2013

c: 2014-2020

d One-sided proportion test for proportion of female and male patients in each group

e Pearson’s chi-square test (two-sided) for comparison of both female and male patient proportions between groups

f Wald Wolfowitz test (one-sided) for female and male patients’ median age in each/between groups

g Mann-Whitney test (one-sided) for female and male patients’ median age between groups

**Table 3:** Patients’ age category distribution of acetaminophen poisonings

Age Category	Group 1 ^a^ N (%)	Group 2 ^b^ N (%)	Group 3 ^c^ N (%)	G1 vs G2 p-value*	G1 vs G3 p-value*	G2 vs G3 p-value**
**Newborn/toddler**	9 (2.2)	22 (2.5)	27 (1.6)	<0.001	<0.001	<0.001
**Preschool**	113 (27.9)	110 (12.5)	59 (3.6)			
**School**	14 (3.5)	6 (0.7)	11 (0.7)			
**Adolescents**	132 (32.6)	330 (37.4)	793 (48.3)			
**Young adult**	114 (28.1)	355 (40.2)	637 (38.8)			
**Adult**	21 (5.2)	56 (6.3)	108 (6.6)			
**Elder**	2 (0.5)°	3 (0.3)	7 (0.4)			
N= 2,929						

* Pearson’s chi-square test (two-sided)

** Exact Fischer’s tests (two-sided)

a: 2001-2007

b: 2008-2013

c: 2014-2020

**Table 4:** Comparisons between patients` gender and poisoning intent for the three groups

	Group 1^a^			Group 2^b^			Group 3^c^					
	A	I	I vs A*	A	I	I vs A*	A	I	I vs A*	G1 vs G2	G1 vs G3**	G2 vs G3**
**Male**	77	38	≈ 1	68	128	<0.001	65	286	<0.001	<0.001	<0.001	<0.001
**Female**	143	147	0.430	115	571	<0.001	109	1,182	<0.001	<0.001	<0.001	<0.001
**Total**	220	185	0.954	183	699	<0.001	174	1,468	<0.001	<0.001	<0.001	<0.001
M vs F*	<0.001	<0.001		<0.001	<0.001		<0.001	<0.001				
Gender vs Intent **	0.002			<0.001			<0.001					
**OR**	2.083			2.638			2.465					
N= 2,929												

A Accidental poisoning I Intentional poisoning OR Odd ratio (1= female and accidental poisoning)

*One-sided proportion test p-value

**Pearson chi-square test (two-sided) p-value

a: 2001-2007

b: 2008-2013

c: 2014-2020

**Table 5:** Multivariate logistic regression model

Coefficients	Estimate	Std. Error	z-value	Pr(>|z|)^a^
(Intercept)	-2.911	0.355	-8.211	<0.001
Age	0.155	0.023	6.879	<0.001
Female	1.355	0.335	4.045	<0.001
Group 2	-0.745	0.391	-1.906	0.057
Group 3	0.883	0.350	2.524	0.012
Age*Female	-0.048	0.022	-2.180	0.029
Age*Group 2	0.168	0.026	6.507	<0.001
Age*Group 3	0.088	0.023	3.895	<0.001

^a^ Wald test (two-sided)
